# Simple Organic Nitrogen Ligand Unlocks Perchlorate Reduction at Neutral pH

**DOI:** 10.1021/acscentsci.6c01255

**Published:** 2026-07-30

**Authors:** Jaehyeong Park, Jong Kwon Choe

**Affiliations:** † Dept. of Civil, Urban and Environmental Engineering and Institute of Construction and Environmental Engineering, 26725 Seoul National University, Seoul 08826, Republic of Korea; ‡ Institute of Engineering Research, Seoul National University, Seoul 08826, Republic of Korea

## Abstract

A simple diamine ligand transforms ruthenium nanoparticles into the first catalyst for rapid perchlorate reduction at neutral pH.

## Graphical abstract

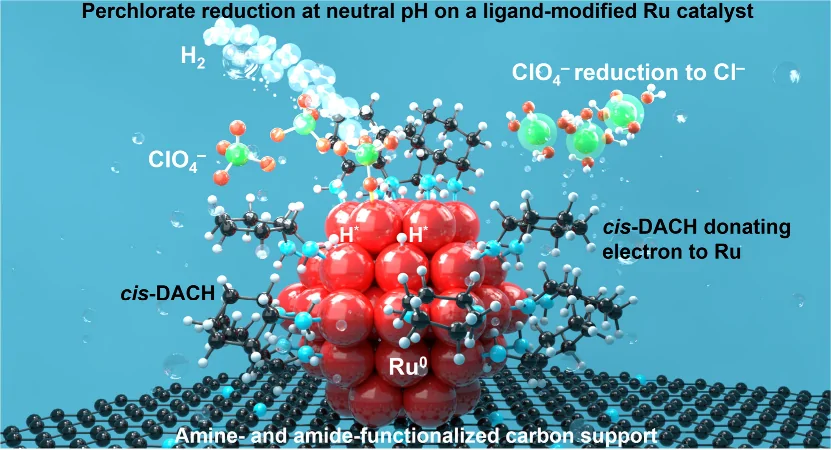

PPerchlorate (ClO_4_^–^), characterized by its chemically inert tetrahedral structure, is a recalcitrant oxyanion that has been widely detected in surface waters, groundwaters, soils, food, and even on other planets.^1^ Its threat to human health, mainly related to disruption of thyroid hormone production, has driven regulators worldwide to tighten drinking water standards.^2^ Thus, practical technologies aimed at reductive transformation of ClO_4_^–^ into innocuous products have been widely investigated via biological and chemical treatment.^3^ However, cleaving the four Cl–O bonds under mild conditions in real water-treatment systems has remained a challenge. In a recent issue of *ACS Central Science*, Liu, Gao, and co-workers report a straightforward yet effective solution: adding an inexpensive aliphatic diamine, *cis*-1,2-diaminocyclohexane (*cis*-DACH), to Ru nanoparticles to create a catalyst that rapidly reduces ClO_4_^–^ at pH 7 and room temperature.^4^

The challenge of catalytic ClO_4_^–^ reduction has a long history. Heterogeneous systems pairing Re with Pd have achieved impressive turnover frequencies, but only at acidic pH.^4,5^ In these catalysts, ClO_4_^–^ is reduced through sequential oxygen atom transfer (OAT): an odd-valence oxorhenium species (Re^V^ or Re^I^) abstracts an oxygen atom from ClO_4_^–^ to give ClO_3_^–^ and a higher valence of Re oxo species, which is then rejuvenated by atomic hydrogen generated through Pd-catalyzed dissociation of H_2_.^6^ Repeated OAT cycles ultimately convert ClO_4_^–^ to Cl^–^. Likewise, Re and Mo oxo complexes inspired by perchlorate reductase, when combined with Pd/C, perform remarkably well, yet are similarly optimized only under acidic conditions.^7,8^ This is because an acidic pH is mechanistically essential for the proton-assisted OAT that drives these catalysts. Above pH 4, the H^+^-dependent OAT pathway falters, and reactivity of these catalysts also collapses as the ligand–metal complex sites decompose.

The authors abandon this paradigm. Rather than engineering an OAT redox cycle, they focus on Ru^0^ nanoparticles, which are already known to exhibit unique reactivity in both reduction and oxidation reactions,^9,10^ and modulate their electronic properties through two types of nitrogen doping. The first strategy is startlingly simple: an organic nitrogen ligand is dissolved in water and stirred with a Ru catalyst under 1 atm H_2_. Among a series of nitrogen ligands screened, *cis*-DACH exhibited the highest turnover frequencies, accelerating ClO_4_^–^ reduction on the Ru/C catalyst up to 80-fold (Figure 1a,b).

The significance of this work is that a dramatic enhancement in catalytic activity arises not from fabricating a metal oxo complex, but from stirring an inexpensive, saturated diamine into a suspension of Ru nanoparticles.

Second, an amine- and amide-functionalized carbon support (NC), prepared by urea nitriding, provided a further 5-fold increase in reactivity. Notably, the resulting [*cis*-DACH]­Ru/NC catalyst effectively reduces ClO_4_^–^ at pH 7 and 20 °C, representing a breakthrough in heterogeneous catalysis studies for ClO_4_^–^ reduction (Figure 1c).

**Figure 1:**
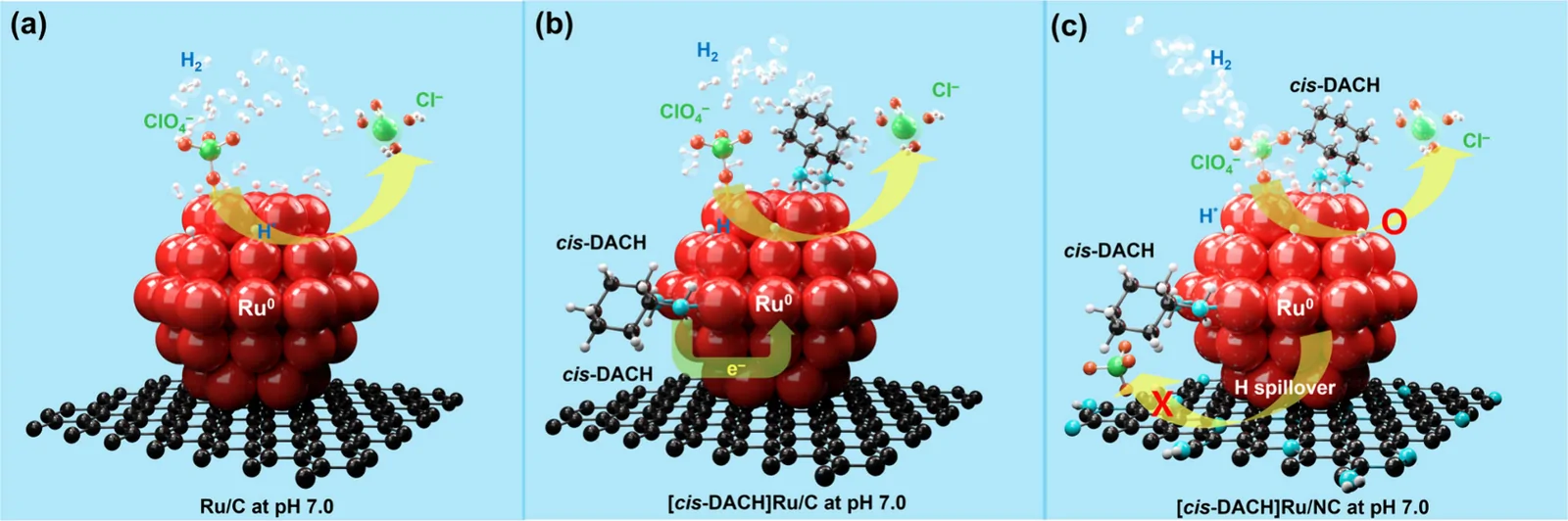
Ru catalyst systems for perchlorate (ClO_4_^–^) reduction. (a) Ru on carbon support (Ru/C), (b) *cis*-DACH-coordinated Ru/C catalyst ([*cis*-DACH]­Ru/C), in which electron donation from *cis*-DACH to Ru accelerates ClO_4_^–^ reduction, (c) [*cis*-DACH]­Ru/NC, in which two nitrogen sources, the *cis*-DACH ligand and an amine/amide functionalized carbon support (NC), together generate an electron-enriched Ru^0^ surface that binds and reduces ClO_4_^–^ more readily, converting ClO_4_^–^ to Cl^–^ with H_2_ at pH 7 and 20 °C, without acid.

To elucidate the underlying mechanisms, the authors performed X-ray photoelectron spectroscopy (XPS), diffuse-reflectance infrared Fourier transform spectroscopy (DRIFT), CO-pulse chemisorption, and kinetic modeling. XPS and DRIFT results consistently indicate an electronic effect: nitrogen coordination shifted the Ru 3p_3/2_ binding energy to lower values and red-shifted the CO stretching bands by 8–26 cm^–1^, both of which signify an electron-enriched Ru^0^ surface.

In essence, the electron donation from the cis-DACH ligand to the Ru^0^ surface accelerated the reduction of ClO_4_^*–*^,which has long been kinetically inert toward catalytic reduction

(Figure 1c). Interestingly, CO-pulse chemisorption testing further revealed that *cis*-DACH occupied approximately 70% of the surface Ru atoms; nevertheless, the reactivity of the remaining exposed Ru sites, where direct interaction between ClO_4_^–^ and Ru is essential, increased 31-fold. Notably, ClO_4_^–^ appears unable to be reduced by atomic hydrogen that spills over from Ru to distant sites on the NC support (Figure 1c). Similarly, Langmuir–Hinshelwood kinetic modeling unveiled the synergistic roles of *cis*-DACH and the NC support: *cis*-DACH enhanced both the adsorption equilibrium constant and the surface reduction rate constant, whereas the NC support further increased the rate constant for adsorbed ClO_4_^–^. This study also demonstrated the stability of the catalyst and its applicability to real water matrices. Because the saturated *cis*-DACH ring contains no unsaturated bonds susceptible to hydrogenation, [*cis*-DACH]­Ru/NC did not undergo gradual deactivation under continuous H_2_-fed treatment. The catalyst showed robustness in maintaining its activity in real water-treatment conditions, including repeated use through ten daily spikes of concentrated ClO_4_^–^, exposure to air, and the presence of competing oxyanions (i.e., NO_3_^–^) in both a synthetic ion-exchange regenerant brine and real tap water.

Several questions and implications remain. For example, the dissolved fraction of *cis*-DACH (∼41%) would need to be managed in full-scale reactors, although the authors propose practical solutions including capture by cation exchange resin and hydrophobic modification of the ligand.

More broadly, this work establishes a catalyst design principle in which organic molecular ligands can serve as tunable electronic modifiers of metal nanoparticle catalysts, achieving catalytic reduction of kinetically inert perchlorate at neutral pH.

By bypassing the acidic requirement that constrained previous perchlorate reduction catalysts, Liu, Gao, and co-workers have addressed a major obstacle to practical application. Furthermore, the simplicity and versatility of the ligand–metal nanoparticle catalyst design extend beyond oxyanions: the same design principle may prove applicable to halogenated organic contaminants, including organofluorine compounds, whose C–F bonds are similarly resistant to reductive cleavage.^11^ This work is therefore expected to inform a new generation of catalyst designs for recalcitrant pollutants in water, from contaminated drinking waters to high-salinity waste brines.
